# Crashworthiness of Foam-Filled Cylindrical Sandwich Shells with Corrugated Cores

**DOI:** 10.3390/ma16196605

**Published:** 2023-10-09

**Authors:** Pengbo Su, Bin Han, Yiming Wang, Hui Wang, Bo Gao, Tian Jian Lu

**Affiliations:** 1 Xi’an Institute of Space Radio Technology, Xi’an 710100, China; su_pengbo@126.com (P.S.); wangyiming0920@126.com (Y.W.); 13519122235@139.com (H.W.); gaob_2004@163.com (B.G.); 2 School of Mechanical Engineering, Xi’an Jiaotong University, Xi’an 710049, China; 3 State Key Laboratory of Mechanics and Control of Mechanical Structures, Nanjing University of Aeronautics and Astronautics, Nanjing 210016, China; tjlu@nuaa.edu.cn; 4 Nanjing Center for Multifunctional Lightweight Materials and Structures (MLMS), Nanjing University of Aeronautics and Astronautics, Nanjing 210016, China

**Keywords:** foam-filled corrugated sandwich cylindrical shells, coupling strengthening effect, energy absorption, theoretical model

## Abstract

Inspired by material hybrid design, novel hybrid sandwich shells were developed by filling a corrugated cylindrical structure with aluminum foam to achieve higher energy absorption performance. The crushing behavior of the foam-filled corrugated sandwich cylindrical shells (FFCSCSs) was investigated using theoretical and numerical methods. Numerical results revealed a significant enhancement in the energy absorption of FFCSCSs under axial compression, showcasing a maximum specific energy absorption of 60 kJ/kg. The coupling strengthening effect is highly pronounced, with a maximum value of ${\bar{F}}_{c}/\bar{F}$ reaching up to 40%. The mechanism underlying this phenomenon can be approached from two perspectives. Firstly, the intrusion of folds into the foam insertions allows for more effective foam compression, maximizing its energy absorption capacity. Secondly, foam causes the folds to bend upwards, intensifying the mutual compression between the folds. This coupling mechanism was further investigated with a focus on analyzing the influence of parameters such as the relative density of the foam, the wall thickness of the sandwich shell, and the material properties. Moreover, a theoretical model was developed to accurately predict the mean crushing force of the FFCSCSs. Based on this model, the influence of various variables on the crushing behavior of the structure was thoroughly investigated through parametric studies.

## 1. Introduction

Researchers have consistently aimed to design high-performance protective equipment and enhance the crashworthiness of various modes of transportation to reduce injuries and property damage resulting from collisions while achieving lightweight designs. Thin-walled shells are widely employed as collision-resistant structures due to their high energy absorption efficiency, reliability, and low manufacturing cost [1]. During collisions, thin-walled shells absorb kinetic energy through significant plastic deformation, safeguarding public safety and protecting property; examples of such shells include energy-absorbing boxes in cars, bumpers in high-speed trains, and crash-resistant landing gears in helicopters. Comprehensive research has been conducted on the energy absorption capacity of single-cell, multicell, and foam-filled shells. Single-cell tubular structures, such as circular, square, and polygonal tubes, have been extensively studied. Corresponding theoretical models for estimating the energy absorption capacity of these structures have been gradually established. When subjected to axial compression, single-cell tubular structures typically exhibit three collapse modes: progressive mode, global mode, and transition mode [2,3]. The progressive collapse mode is the primary focus of most studies because it exhibits stable deformation processes and possesses higher energy absorption efficiency. In the case of circular tubes, Guillow et al. divided the progressive collapse mode into axisymmetric mode, nonsymmetric mode, and mixed mode [4]. Alexander [5] derived an approximate theory to estimate the collapse load specifically for the axisymmetric mode. For square tubes, Weirzbickihe and Abramowicz [6,7] proposed a fundamental folding mode known as the “super folding element” based on their observation of the folding deformation process. This mode was utilized to predict the mean crushing force. Subsequently, the “super folding element” was extended to polygonal tubes with arbitrary interior angles [8]. Triangular tubes [9,10,11], hexagonal tubes [12,13,14,15], octagonal tubes [16], nonconvex multicorner tubes [17,18], and star-shaped tubes [15,19] have been extensively investigated based on this work. It is demonstrated that energy dissipation primarily occurs at horizontal plastic hinges. Consequently, increasing the number of folds would significantly improve the energy absorption capacity of tubular structures.

To further enhance energy absorption, researchers proposed tubular structures filled with foam or honeycomb cores. Corresponding studies indicated that filled tubular structures exhibited significantly more folds than unfilled ones, resulting in higher energy absorption efficiency [20]. Reid et al. [21,22] conducted a series of static and dynamic experiments on circular and square tubes filled with polyurethane foam. Their findings revealed that the specific energy absorption of foam-filled structures was twice as high as that of nonfilled structures. Compared to polyurethane foam, metal foam, specifically aluminum foam, exhibits higher platform stress levels. With the commercial preparation process for aluminum foam gradually maturing, subsequent researchers conducted extensive studies on aluminum-foam-filled tubes with various cross sections, including square tubes [20,23,24,25], circular tubes [26,27,28], and polygonal tubes [23,29]. Furthermore, the potential of honeycomb-filled tubular structures in energy absorption was thoroughly examined by Hussein et al. [30] and Yin [31]. These studies effectively demonstrated the advantages of honeycomb filling to enhance energy absorption capabilities. Recently, novel fillings made of cellular materials were proposed based on the continuous development of configuration and preparation processes. These materials include functionally graded foam [32,33,34,35], auxetic foam [36], composite foam [37,38], ex situ aluminum foam [39], and liquid nanofoam [40].

To further improve the energy absorption capacity of tubular structures, researchers designed sandwich tubular structures filled with cellular materials, such as foams and honeycombs. The advantageous energy absorption capabilities of these sandwich structures were demonstrated by Seitzberger et al. [23], Li et al. [41], Zhang et al. [42], Zheng et al. [43], Gao et al. [44], Djamaluddin et al. [45], and Goel [46]. These investigations revealed that the sandwich structures exhibited a higher mean crushing force due to the coupling effect between the face sheets and the filling materials. Meanwhile, research findings revealed that sandwich structures featuring two-dimensional (2D) corrugated or honeycomb cores offered superior weight reduction and design benefits, as evidenced by studies [47,48,49,50,51,52]. In our previous work, the energy absorption capacity of corrugated sandwich shells was investigated through a combined experimental, theoretical, and numerical approach [53,54].

Moreover, due to the interconnected nature of the corrugated channels, some researchers filled these channels with cellular materials such as foam and aluminum honeycomb to enhance structural performance. Foam-filled corrugated sandwiches were designed and fabricated by Yan et al. [55] and Han et al. [56]. Their work revealed that the energy absorption performance of these structures under out-of-plane compression surpassed the combined energy absorption of the hollow corrugation and the foam individually, attributed to the coupling effect between the foam and the corrugated core. Similar coupling effects were also observed in honeycomb–corrugate hybrid structures [57], ceramic–corrugate hybrid structures [58,59,60], and other hybrid sandwich structures [61,62,63,64].

To further enhance the energy-absorbing properties of the tubular structure, a novel hybrid sandwich shell was proposed by incorporating aluminum foam into a corrugated sandwich cylindrical shell. To characterize the crushing behavior, the finite element (FE) method was employed, and its accuracy was verified using experimental data in reference [53]. The energy absorption of the FFCSCS under axial compression was investigated through numerical simulations. The collapse behavior and folding modes were analyzed, and the coupling strengthening mechanism between foam and shell wall was explored. Based on FE simulations, a theoretical model was developed to predict the mean crushing force of FFCSCSs. Parametric analysis was conducted using the theoretical model to explore the influence of different parameters on the coupling strengthening effect. This paper is organized as follows: Section 2 introduces the definition of terminology. Section 3 presents and validates the finite element model. Section 4 provides an analysis of the coupling strengthening effect and its mechanism. Section 5 discusses the influence of wall thickness, material, and foam density on the energy absorption performance of FFCSCSs based on finite element analysis. Section 6 presents a theoretical model for predicting the mean crushing force and conducts a parametric study based on the mechanistic model.

## 2. Terminology Definition in the Crushing Process

**Figure 1:**
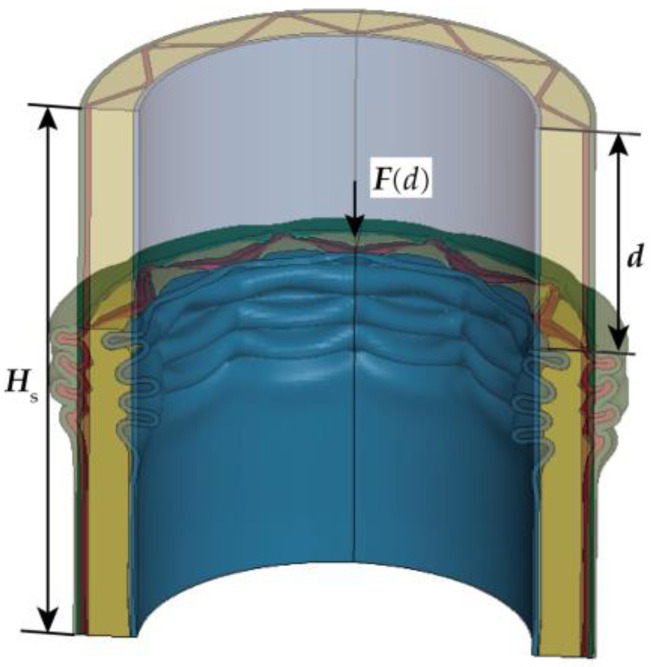
The FFCSCS is under crushing process.

This section presents the relevant physical quantities and their definitions used to describe the crushing process and assess energy absorption characteristics of cylindrical shells under axial compression. As illustrated in Figure 1, *H*_s_ represents the initial height of the cylindrical shell, *d* is employed to signify the compressive displacement of the structure, and *F* characterizes the corresponding crushing force within the structure. 

The maximal value of *F* within the interval from 0 to *d* is denoted as *F*_max_(*d*). Simultaneously, the energy absorption within this stage is defined as *E*(*d*), which can be expressed as

(1) $$E(d)=\int_{0}^{d}F(d)dx$$

Building upon this, the mean crushing force within the interval from 0 to *d* is denoted as $\bar{F}(d)$, which can be expressed as follows:

(2) $$\bar{F}(d)=\frac{E(d)}{d}$$

Meanwhile, *T*_E_ (*d*) represents the energy absorption efficiency of the structure, and its expression is given as follows:

(3) $$T_{E}(d)=\frac{E(d)}{F_{\max}(d)H_{s}}$$

When under compression, *T*_E_ (*d*) exhibits a trend where it initially increases with the increasing value of *d* and subsequently decreases [20]. During this progression, *T*_E_ (*d*) reaches a maximum value, corresponding to the peak energy absorption efficiency of the structure [20]. The compression displacement at which *T*_E_ (*d*) reaches its maximum value is defined as *d*_max_ [20]. The values of $\bar{F}(d)$ and *F*_max_(*d*) at this specific compression displacement *d*_max_ are subsequently designated as the ultimate mean crushing force $\bar{F}$ and maximum crushing force *F*_max_ of the structure. Expanding on this concept, the crushing force efficiency, labeled as *A*_E_, is defined as $\bar{F}/F_{max}$. In this study, SEA (specific energy absorption) represents the energy absorbed per unit mass by the structure during the compression failure process, and its expression is as follows:

(4) $$\mathrm{SEA}=\frac{E(d_{\max})}{M_{s}}$$

where *E*(*d*_max_) signifies the energy absorption of the structure at *d*_max_ and *M*_s_ represents the mass of the structure.

For an ideally energy-absorbing structure, the objective is to maximize energy absorption within a specified compression displacement while keeping the crushing force during compression within acceptable limits. Simultaneously, the structure should possess lightweight characteristics. Translating these prerequisites into energy absorption parameters, the goal is to attain higher values for $\bar{F}$, SAE, and *A*_E_ while striving for a lower value of *F*_max_.

## 3. Finite Element Modeling

### 3.1. Descriptions of the Geometric Model

**Figure 2:**
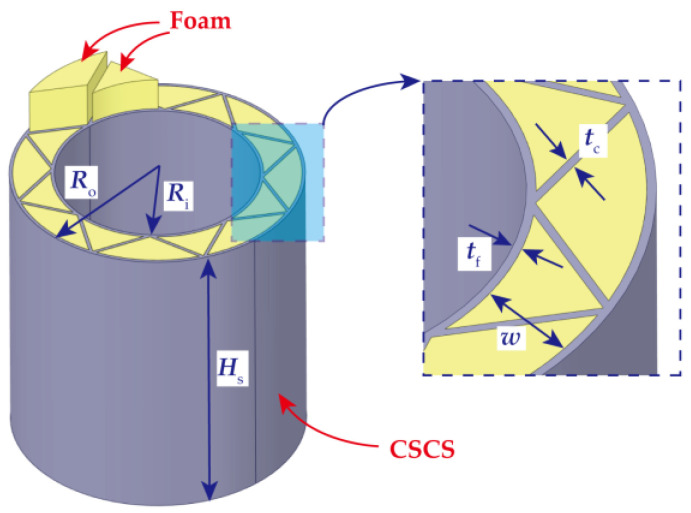
Geometric schematic of foam-filled corrugated sandwich cylindrical shells.

Critical geometric parameters of the FFCSCS are presented in Figure 2, including inner radius (*R*_i_), outer radius (*R*_o_), number of corrugations (*N*), thickness of the inner and outer face sheets (*t*_f_), thickness of the corrugated core (*t*_c_), width of the corrugated core (*w*), and height of the shell along *z* direction (*H*_s_). In the present study, *t*_c_ = *t*_f_ = *t* is specifically emphasized. The mass of the FFCSCS can be determined as follows:

(5) $$M_{s}=2tH\rho_{s}\left[\pi (R_{i}+R_{o})+Nw\right]+\rho_{f}H\left[\pi (R_{o}^{2}-R_{i}^{2})-Nwt\right]$$

where the mass of FFCSCSs is divided into two components: the first component represents the corrugated sandwich cylindrical shell’s mass, and the second component represents the mass of the filled foam.

### 3.2. FE Model

**Figure 3:**
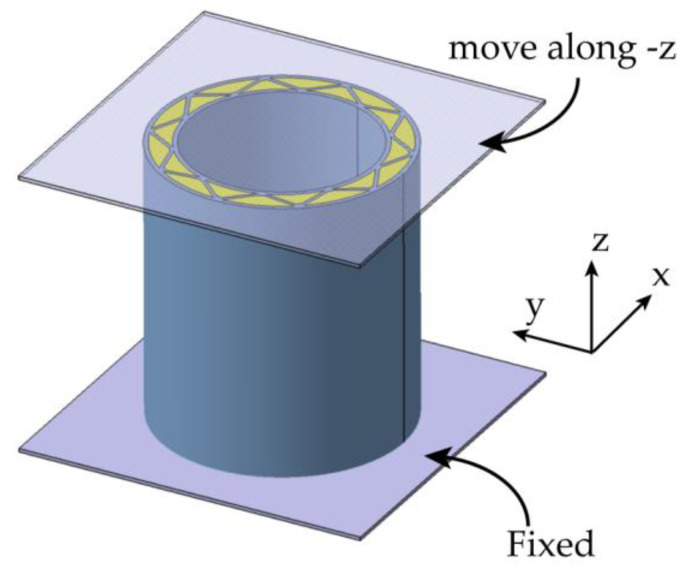
The finite element model of the FFCSCS under axial compression condition.

The finite element (FE) analysis in this study was performed using the commercial finite element software LS-DYNA 971, employing its explicit algorithm. The FE model is given in Figure 3. Both the upper and lower plates were modeled as rigid bodies and simulated in LS-DYNA utilizing the *MAT_RIGID card. To impose appropriate boundary conditions, the lower plate was in its position, while the upper plate was constrained to have all degrees of freedom except for translational motion in the *z* direction. A displacement load was applied to the upper plate in the negative *z*-axis direction. The loading rate of 1 m/s was employed. At this rate, the kinetic energy within the structure represented less than 1% of the total energy, leading to an approximately quasi-static process.

In the finite element model, the corrugated sandwich shell, aluminum foam, and rigid plates were meshed with SOLID164 solid elements. The corrugated sandwich shell was meshed with a minimum element size of 0.2 mm × 0.2 mm × 0.2 mm. This mesh size ensured that at least three layers of elements were present along the wall thickness direction of the shell, allowing for an appropriate resolution. For the aluminum foam, a mesh size of 0.25 mm × 0.25 mm × 0.25 mm was used. The upper and lower pressure plates, treated as rigid bodies, were meshed with a size of 0.3 mm × 0.3 mm × 0.3 mm. Mesh sensitivity study showed that further mesh refining did not yield improvements in the accuracy of the simulation results. Therefore, the current mesh size achieves a balance between computational accuracy and efficiency.

During the simulation, the contact interactions between the upper or lower plates and FFCSCS were defined by the *CONTACT_AUTOMATIC_SURFACE_TO_SURFACE card. To model the internal self-contact within the composite cylindrical sandwich shell structure, the *CONTACT_AUTOMATIC_SINGLE_SURFACE card was employed. To simulate the bonding relationship between foam and corrugated sandwich shell walls, the *CONTACT_AUTOMATIC_SURFACE_TO_SURFACE_TIEBREAK card was utilized. When significant normal and tangential stress occurred at the interface between the adhesive interfaces, this bonding relationship automatically degraded to *CONTACT_AUTOMATIC_SURFACE_TO_SURFACE according to the following degradation criterion [65]:

(6) $${\left(\frac{\left|\sigma_{n}\right|}{\mathrm{NFLS}}\right)}^{2}+{\left(\frac{\left|\sigma_{s}\right|}{\mathrm{SFLS}}\right)}^{2}\geq 1$$

where *σ*_n_ and *σ*_s_ denote the normal and tangential stresses between the adhesive interfaces. NFLS and SFLS are the tensile and shear strengths of the bonding material. These strengths are determined based on the Loctite Hysol E-120HP two-component epoxy adhesive from Henkel, with measured values of 41 MPa for NFLS and 33 MPa for SFLS [66].

### 3.3. Material Properties

**Figure 4:**
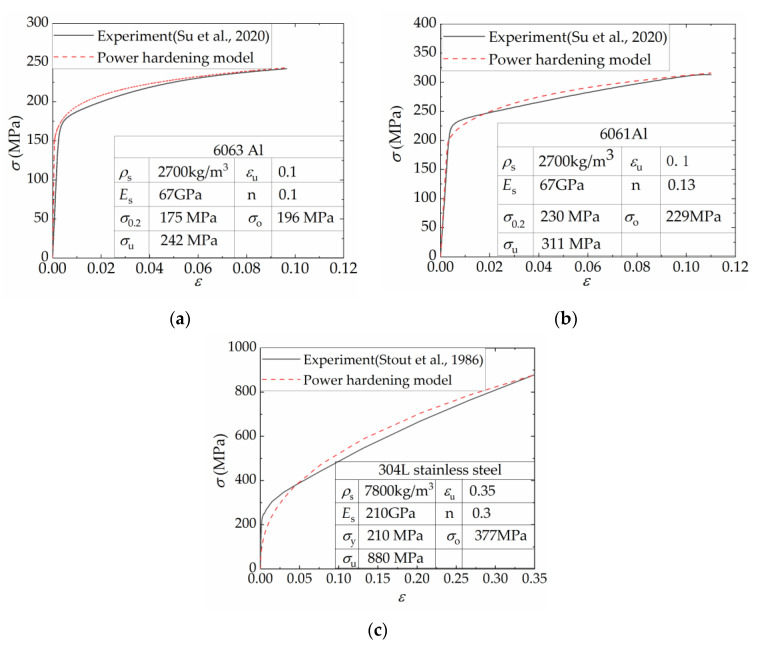
Material properties and corresponding power-hardening model of parent materials for corrugated cores and face sheets: (**a**) 6063 Al [54]; (**b**) 6061Al [54]; (**c**) 304L stainless steel [67].

The materials considered for the face sheets and core in this study are 6063Al, 6061Al, and 304L stainless steel, respectively. The real stress–strain curves measured in experiments are sourced from references [54,67], depicted in Figure 4. The solid black lines in the figure represent the experimentally obtained data, with the corresponding material parameters denoted as follows: density (*ρ*_s_), elastic modulus (*E*_s_), yield strength (*σ*_0.2_ or *σ*_y_), ultimate strength (σ_u_), and ultimate strain (*ε*_u_). The hardening behavior of the materials is described by a power-hardening model represented by the equation $\sigma =\sigma_{u}{\left(\varepsilon /\varepsilon_{u}\right)}^{n}$, which is depicted by the red dashed line in Figure 4. The parameter *n* represents the power-law-hardening exponent, which is determined through fitting to the experimental data. *σ*_o_ represents the flow stress, considering the strain-hardening effects of the metal material. In the case of the power-law-hardening model, *σ*_o_ can be expressed as [24]

(7) $$\sigma_{o}=\sqrt{\frac{\sigma_{u}\sigma_{y}}{1+n}}$$

The materials mentioned above were considered isotropic elastic–plastic solids with isotropic hardening in the finite element analysis. The Mises yield rule and J2 flow law were employed. The *MAT_PIECEWISE_LINEAR_PLASTICITY intrinsic model was utilized for the three materials in LS-DYNA, while the material’s dynamic strengthening effect was not considered.

This research investigated the effects of varying relative densities of aluminum foam on the coupled strengthening effect of FFCSCSs. To mitigate the errors arising from uncertainties in the aluminum foam processing process and substrate material, a theoretical model developed by Hanssen et al. [68] was used to derive the material parameters of foam material as follows:

(8) $$\sigma_{p}=720{\bar{\rho }}_{f}{}^{2.33}$$

(9) $$E_{p}=330{\bar{\rho }}_{f}{}^{2.45}$$

where ${\bar{\rho }}_{f}$ represents the relative density of the foam, *σ*_p_ denotes the yield strength of the foam in MPa, and *E*_p_ refers to the modulus of elasticity of the foam in GPa.

The stress–strain curve of the foam after yielding was also obtained using the equation given by Hanssen et al. [68]:

(10) $$\sigma =\sigma_{p}+42{\bar{\rho }}_{f}{}^{1.42}\frac{e}{e_{D}}+251{\bar{\rho }}_{f}\ln\left[\frac{1}{1-{\left(e/e_{D}\right)}^{\beta }}\right]$$

where *e* is the engineering strain of the foam, *σ* is the compressing stress of the foam, $\beta =1/(0.1+15.7{{\bar{\rho }}_{f}}^{3})$, and $e_{D}=1-{\bar{\rho }}_{f}$. This formula applies to the relative density of the foam ranging from 0.05 to 0.2.

**Figure 5:**
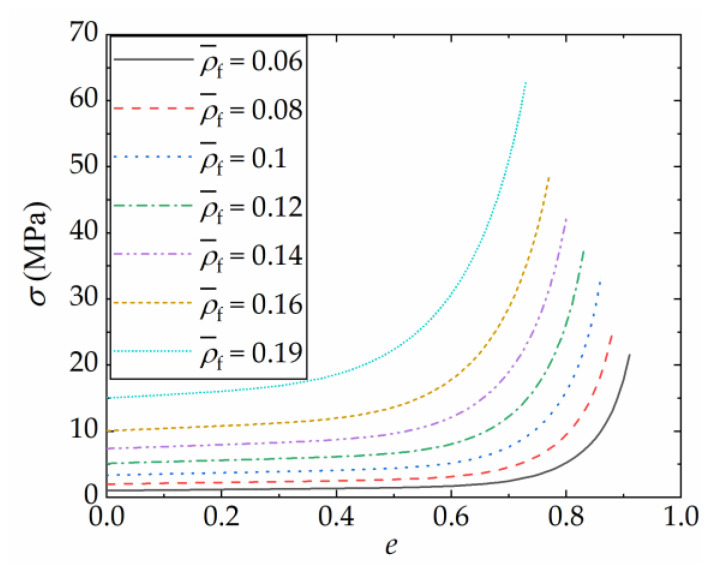
Material properties of the filled foams with different relative densities (${\bar{\rho }}_{f}$).

Figure 5 displays the compressive stress–strain curves of seven relative density foams obtained using the equations mentioned above. The influence of these foams on the coupling enhancement effect of the FFCSCSs will be examined and discussed. In LS-DYNA, the foam material was defined using *MAT_CRUSHABLE_FOAM and identified as Material Type 63 in LS-DYNA. This material model requires the specification of mass density, Young’s modulus, Poisson’s ratio, and a load curve. The load curve encompasses both the plateau and densification stages that occur after the foam material reaches its yield point. It should be noted that in the finite element analysis conducted in this paper, the failure of the foam material, specifically in terms of fracture, was not taken into consideration.

### 3.4. Validation against Experiments

**Figure 6:**
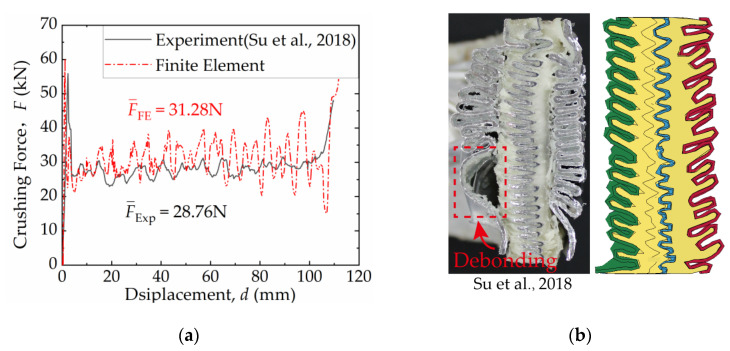
Comparison between experiments [53] and FE results of PMI foam-filled 1060 Al sandwich cylindrical shell: (**a**) force–displacement curves; (**b**) final collapse mode.

In reference [53], preliminary axial compression tests were conducted on the FFCSCS. The experimental findings demonstrated a significant coupling enhancement effect of the FFCSCS when subjected to axial compression, in contrast to the independent foam and shell components. Figure 6 presented the comparison between the experimental and numerical results. In the experiment [53], the mean crushing force of the structure was 28.76 N, and the mean crushing force obtained through simulation in this work was 31.28 N. The excellent agreement between the two values indicates that the simulation approach employed in this study effectively characterizes the energy absorption characteristics of the structure. Furthermore, the final collapse mode captured from the FE results closely resembles the experimental photo, except for localized debonding observed in the experiments.

## 4. Numerical Results

Based on FE simulations, a comprehensive study of the crushing behavior of the FFCSCS, encompassing the crushing process, the coupling enhancement effect, and its underlying mechanisms, is presented in this section.

### 4.1. Crushing Process

A representative structure is selected for a detailed analysis of the crushing process of FFCSCSs under axial compression. The shell material of this structure is 6063 aluminum, the wall thickness of the shell component is 0.8 mm, and the foam has a relative density of 0.1. This representative structure is labeled as 6063-08-01, signifying the shell material (6063), the shell-wall thickness (08), and the foam relative density (01).

**Figure 7:**
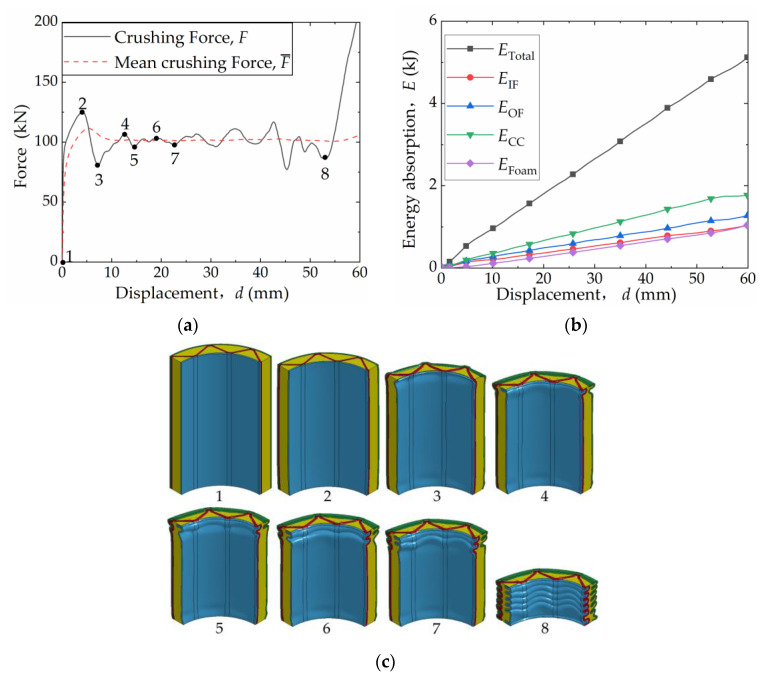
Crushing process and energy absorption of FFCSCS 6063-08-01: (**a**) force–displacement curves; (**b**) energy absorption–displacement curves; (**c**) collapse configurations with labels corresponding to those marked in the force–displacement curves.

Figure 7a depicts the crushing force–compression displacement (*F-d*) curve for the 6063-08-01 structure. Figure 7b presents the energy absorption–compression displacement curve (*E-d* curve). Figure 7c shows the deformation configurations corresponding to the peak and trough values of the *F-d* curve (Only 1/4 of the structure is shown to facilitate the observation of internal deformation).

Initially, the *F-d* curve exhibits a linear increase, indicating the structure is primarily in the elastic stage. Subsequently, a bifurcation point emerges on the *F-d* curve, signifying the transition from the linear–elastic stage to the nonlinear zone, where the crushing force *F* continues to increase. At point 2 on the *F-d* curve, the first fold in the structure begins to form and reaches its peak value before sharply declining. As compression progresses from point 2 to point 3, the folding area expands, resulting in a gradual decrease in *F*. From point 3 to point 4, mutual compression occurs within the first fold, causing the *F-d* curve to rise. As the new fold starts to form between point 4 and point 5, the *F-d* curve once again declines. Throughout the compression process, the *F-d* curve alternates between peak and valley values as folds form, expand, and extrude layer by layer. Upon reaching point 8, the curve enters the densification stage, exhibiting a rapid increase.

A clear trend can be observed that the energy absorption (*E*_total_) increases linearly with the increase in compression displacement (*d*). The corrugated core exhibits the highest energy absorption capacity (*E*_cc_), surpassing that of the outer face sheet (*E*_OF_). At smaller *d* values, the inner face sheet absorbs slightly more energy (*E*_IF_) compared to the foam (*E*_Foam_). However, as *d* increases, *E*_IF_ gradually becomes equivalent to *E*_Foam_.

### 4.2. Coupling Enhancement Effect

The coupling strengthening behavior and the strengthening mechanism of the FFCSCS 6063-08-01 are explored here. Additionally, separate simulation analyses are conducted for the two constituents of the FFCSCS: the corrugated core shell (CSCS) and the foam column (foam). This enables the analysis of the energy absorption contributions arising from the coupling strengthening effect. In this study, “foam + CSCS” represents the algebraic sum of the energy absorption characteristics of the two components when analyzed independently and does not represent an actual physical structure.

**Figure 8:**
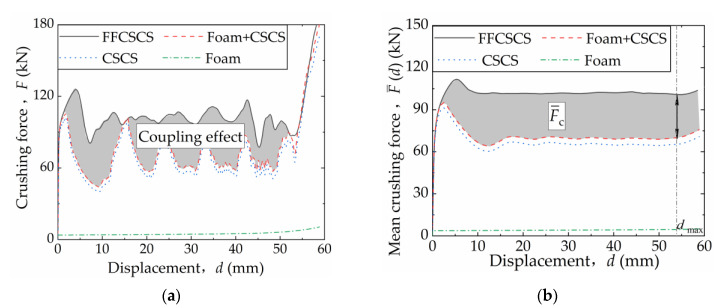
Coupling enhancement effect in FFCSCS 6063-08-01: (**a**) force–displacement; (**b**) mean crushing force–displacement curves.

Figure 8a presents the crushing force–displacement (*F-d*) curves for 6063-08-01 and its individual constituents when subjected to independent compression. The *F-d* curve for the FFCSCS is represented by a solid black line, while those for the independently compressed corrugated sandwich cylindrical shell (CSCS) and foam column are depicted by a dashed blue line and a dotted green line, respectively. The curve for the “Foam + CSCS” combination is shown as a dashed red line. The shaded region between the solid black line and the red dashed line depicts the pronounced coupling strengthening effect observed between the components of the FFCSCS.

Based on the *F-d* curves depicted in Figure 8a, the mean crushing force $\bar{F}\left(d\right)$ for the FFCSCS and its individual components as a function of the compression displacement *d* is calculated and shown in Figure 8b. The shaded region in Figure 8b represents the coupling strengthening effect characterized by $\bar{F}\left(d\right)$, which exhibits a progressive increase with the increasing *d* until it reaches a stable state.

The final mean crushing forces for the FFCSCS, foam, and CSCS are denoted as $\bar{F}$, ${\bar{F}}_{f}$, and ${\bar{F}}_{s}$, respectively. Therefore, the coupling strengthening effect is characterized by the coupling mean crushing force, expressed as ${\bar{F}}_{c}$, and is determined as follows:

(11) $${\bar{F}}_{c}=\bar{F}-{\bar{F}}_{s}-{\bar{F}}_{f}$$

Based on the definitions and compression curves mentioned above, several parameters are calculated for the CSCS, foam, and FFCSCS, including the mean crushing force $\bar{F}$, coupling mean crushing force ${\bar{F}}_{c}$, specific energy absorption SEA, and the crushing force efficiency *A*_E_. The values for these parameters are presented in Table 1. It is evident that in the case of FFCSCS, ${\bar{F}}_{c}$ accounts for 31% of $\bar{F}$, indicating a significant coupling strengthening effect between the components. Furthermore, a comparison between the different configurations reveals that the SEA and *A*_E_ of the FFCSCS are considerably enhanced compared to CSCS and the foam.

### 4.3. Mechanism of Coupling Enhancement

In the preceding section, it is observed that a pronounced coupling strengthening effect exists among the components of FFCSCS 6063-08-01. To further reveal the coupling strengthening mechanism, the energy absorption properties and folding mode of FFCSCS, individual CSCS, and individual foam components are analyzed separately. It is worth mentioning that the individual foam component utilized in the analysis is in the form of a solid cylindrical structure, aligning its height with that of the FFCSCS. Its cross-sectional area encompasses the total area occupied by all foam sections within the corrugated channels.

Table 2 presents the energy absorbed by each component in the FFCSCS, the CSCS, and the foam. It is evident that in the FFCSCS, the corrugated core absorbs the highest amount of energy, followed by the outer face sheet, the inner face sheet, and the foam. Similarly, in the CSCS, the corrugated core absorbs the most energy, followed by the outer and inner face sheets. When comparing the energy absorption of the corresponding components in the FFCSCS and CSCS, it is notable that the corrugated core, outer face sheet, and inner face sheet of the FFCSCS exhibit increased energy absorption. Specifically, the inner face sheet shows a 26% increase, the outer face sheet shows a 14% increase, and the corrugated core shows a 5% increase. Moreover, when comparing the energy absorption of the foam in the FFCSCS with that of an equally massed individual foam column, it is evident that the filled foam in the FFCSCS exhibits a remarkable improvement. The energy absorption of the filled foam is enhanced by 283% compared to that of the individual foam column.

**Figure 9:**
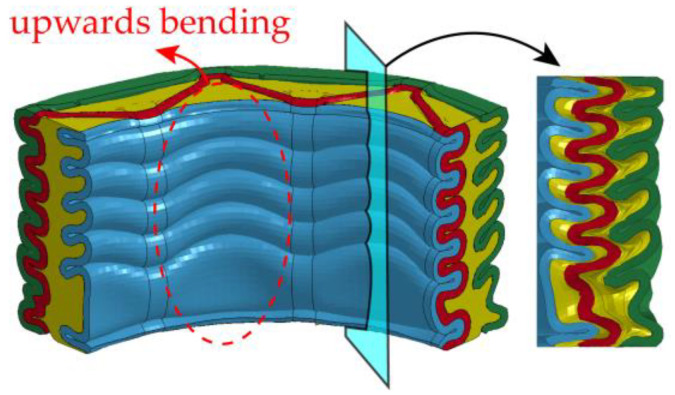
Collapse configuration of the FFCSCS 6063-08-01.

In Figure 9, the collapse mode of FFCSCS 6063-08-01 after compaction is depicted, with a quarter of the structure intercepted to facilitate observation of internal deformation. The collapse mode of FFCSCS 6063-08-01 exhibits an axisymmetric pattern, while independent local folds form along the corrugated core. Upon closer examination, it becomes apparent that shell folds intrude into the foam region during compression. This deformation mode allows for a more thorough foam compression, resulting in increased energy absorption compared to an individual foam column. Moreover, the foam alters the deformation modes of the folds in the corrugated core and face sheets. Figure 9 clearly illustrates the upward bending of the folds in the inner face sheet across all folding layers.

Table 3 provides an overview of the collapse configurations observed in the inner face sheet (IF), outer face sheet (OF), and corrugated core within both the FFCSCS and CSCS structures. In the initial row of the table, the collapse configurations of the IF within both structures are depicted, with the folds in each layer highlighted by a red line. It is evident that in the CSCS, the folds of the IF exhibit minimal deformation in the compression direction. In contrast, in the FFCSCS, all the folds are observed to bend upwards along the compression direction. Moving to the second row of Table 3, a comparative analysis of the collapse mode of the OF is presented.

Similarly, in the FFCSCS, the folds in the OF exhibit bending along the compression direction, whereas the folds in the CSCS display minimal deformation in this direction. The bending deformation of the folds in the FFCSCS results in increased plastic deformation of the material and enhanced interfolding compression. These two factors synergistically contribute to the improved energy absorption properties of the FFCSCS. Furthermore, the collapse mode of the corrugated core, as depicted in the third row of Table 3, exhibits a nearly identical behavior in both the CSCS and FFCSCS. In summary, the coupling effect is more pronounced for the inner and outer face sheets of the FFCSCS, whereas it is comparatively weaker for the corrugated core.

**Figure 10:**
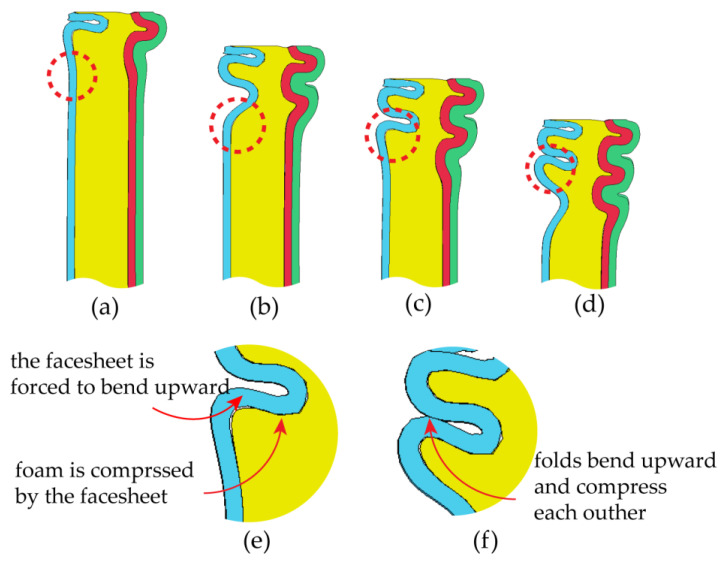
The formation process of folds (enclosed by the red dash circle) in FFCSCS (6063-08-01): (**a**) initial stage; (**b**) beginning of formation; (**c**) bending upwards; (**d**) compressing each other; (**e**) partially enlarged view of (**c**); (**f**) partially enlarged view of (**d**).

To elucidate the observed folding phenomenon in the inner and outer face sheets of the FFCSCS, Figure 10 provides a visual representation of the formation of the second layer of folds within the inner face sheet.

In Figure 10a, the initial stage of the crushing process is depicted, where the formation of the second layer of folds has not yet commenced. As depicted in Figure 10b, the initiation of the second layer of folds begins as the compression displacement increases. With further compression, as shown in Figure 10c, the second layer of folds becomes progressively compressed, resulting in their upward bending along the direction of compression. Moving to Figure 10d, it is evident that the second layer of folds is fully developed, and the folds bend upwards along the compression direction. Additionally, it is observed that the second layer of folds comes into contact with the first layer of folds, giving rise to mutual compression due to the bending deformation.

For a more detailed examination of this process, Figure 10e partially magnifies Figure 10c. It becomes apparent that the foam adjacent to the fold undergoes compression due to the pressure exerted by the face sheet, causing the foam material to flow upwards. This upwards flow of foam material subsequently forces the adjacent region of the face sheet to bend upwards. Similarly, Figure 10f partially enlarges Figure 10d, illustrating how the folds are forced to contact and compress each other. This collapse mode further enhances the energy absorption properties of the structure.

## 5. Discussion

The preceding analysis reveals the coupling strengthening effect and its underlying mechanisms. This section discusses the influence of the foam’s relative density, the wall thickness, and the materials of the corrugated sandwich on the coupling strengthening effect.

### 5.1. Influence of Foam Density on the Coupling Effect

The influence of the relative density of the foam (${\bar{\rho }}_{f}$) on the coupling enhancement effect of the FFCSCSs is investigated in this section. The relative density of the foam ${\bar{\rho }}_{f}$ varies within the range of 0.06 to 0.19 while maintaining a constant shell material (6063 Al) and a shell-wall thickness of 0.8 mm.

**Figure 11:**
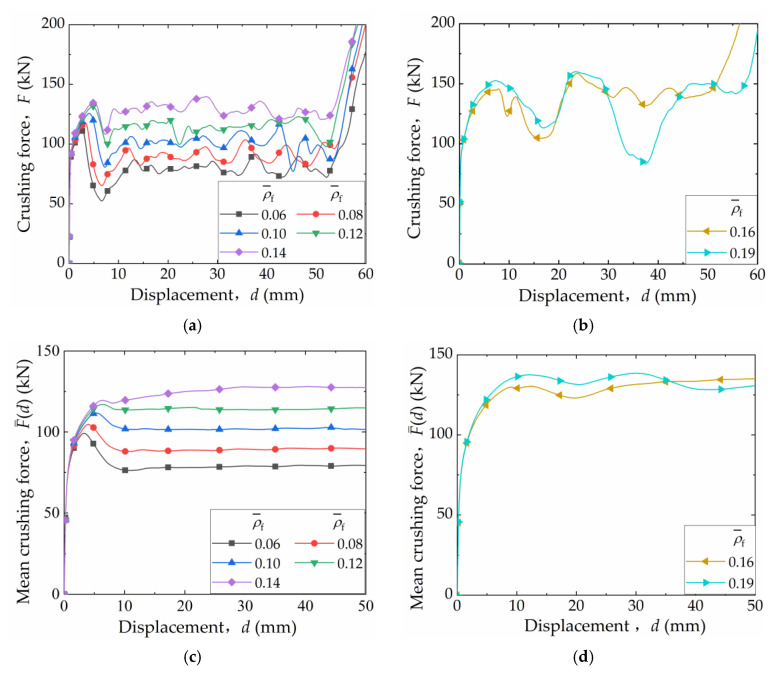
Crushing response of FFSCSCs with different relative foam density ${\bar{\rho }}_{f}$: (**a**) force–displacement curves for ${\bar{\rho }}_{f}$ = 0.06~0.14; (**b**) force–displacement curves for ${\bar{\rho }}_{f}$ = 0.16~0.19; (**c**) mean crushing force–displacement curves for ${\bar{\rho }}_{f}$ = 0.06~0.14; (**d**) mean crushing force–displacement curves for ${\bar{\rho }}_{f}$ = 0.16~0.19.

Figure 11 illustrates the crushing force–compression displacement curves (*F-d* curves) and compression mean crushing force–displacement curves ($\bar{F}$-*d* curves) for FFCSCSs with varying ${\bar{\rho }}_{f}$ ranging from 0.06 to 0.19. In Figure 11a, it is evident that the *F-d* curves for ${\bar{\rho }}_{f}$ ranging from 0.06 to 0.14 exhibit similar patterns. Initially, each curve reaches its peak value, followed by fluctuations around a stable value, and it finally undergoes a rapid rise due to compaction. Notably, both the peak and stable values in the *F-d* curves increase with an increasing value of ${\bar{\rho }}_{f}$. However, in Figure 11b, the shape of the *F-d* curves changes as ${\bar{\rho }}_{f}$ increases to 0.16 and 0.19. The curves display overall fluctuations without any distinct peak or stable values. Moving to Figure 11c, the $\bar{F}$-*d* curves for FFCSCSs with ${\bar{\rho }}_{f}$ values of 0.06 to 0.14 are presented. It is observed that as displacement (*d*) increases, the mean crushing force ($\bar{F}(d)$) also increases and gradually converges to the constant value ($\bar{F}$). Furthermore, it is evident that $\bar{F}$ increases with increasing ${\bar{\rho }}_{f}$. However, in Figure 11d, the $\bar{F}$-*d* curves exhibit a rising and falling pattern with an increase in *d* for structures with ${\bar{\rho }}_{f}$ values of 0.16 and 0.19, without converging to a constant value.

**Figure 12:**
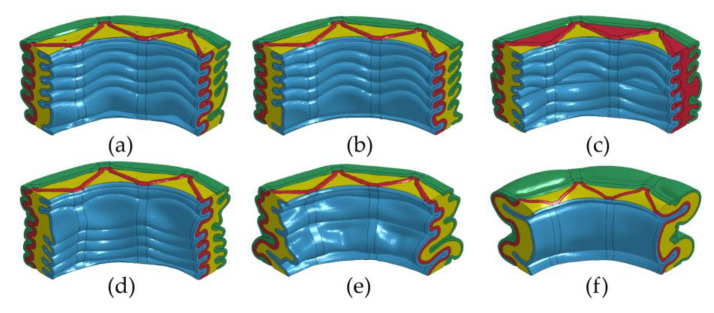
Collapse configuration of FFSCSCs with ${\bar{\rho }}_{f}$ values ranging from 0.08 to 0.19: (**a**) 0.08; (**b**) 0.10; (**c**) 0.12; (**d**) 0.14; (**e**) 0.16; (**f**) 0.19.

Figure 12 illustrates the collapse modes of FFCSCSs with ${\bar{\rho }}_{f}$ values ranging from 0.08 to 0.19. For ${\bar{\rho }}_{f}$ between 0.08 and 0.14, the structures exhibit the progressive folding mode, where the folds occur layer by layer along the compression direction. This phenomenon is depicted in Figure 11a, where the *F-d* curves show fluctuations, indicating the layer-by-layer formation of folds. However, as shown in Figure 12d, when ${\bar{\rho }}_{f}$ reaches 0.14, some of the folds in the FFCSCSs are not fully developed, and a tendency toward global deformation begins to emerge. As ${\bar{\rho }}_{f}$ increases to 0.16 and 0.19, the deformation mode of the structure transitions to a global folding mode, as demonstrated in Figure 12e,f. In this global folding mode, the *F-d* curves no longer exhibit fluctuations around a stable value, as observed in Figure 11b.

Table 4 presents the crushing performance of the FFCSCSs for ${\bar{\rho }}_{f}$ values ranging from 0 to 0.19, where ${\bar{\rho }}_{f}=0$ represents the CSCS. It is evident that the FFCSCSs exhibit significantly higher mean crushing force ($\bar{F}$) and specific energy absorption (SEA) compared to the CSCS. For ${\bar{\rho }}_{f}\leq 0.16$, $\bar{F}$, ${\bar{F}}_{c}$, SEA and *A*_E_ of the FFCSCSs increase with an increasing value of ${\bar{\rho }}_{f}$. However, as ${\bar{\rho }}_{f}$ further increases to 0.19, $\bar{F}$, ${\bar{F}}_{c}$, SEA, and *A*_E_ decrease. This decline can be attributed to the global deformation, as depicted in Figure 12f, when ${\bar{\rho }}_{f}$ exceeds a certain threshold.

As indicated in Equation (2), the mean crushing force of the FFCSCS ($\bar{F}$) is composed of the mean crushing force of the CSCS (${\bar{F}}_{s}$), the mean crushing force of the foam (${\bar{F}}_{f}$), and the coupling mean crushing force (${\bar{F}}_{c}$).

**Figure 13:**
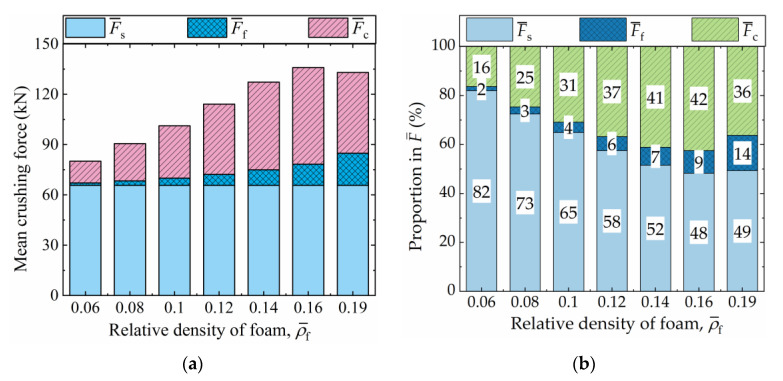
Composition of the mean crushing force ($\bar{F}$) for FFCSCSs with ${\bar{\rho }}_{f}$ = 0.06~0.19: (**a**) absolute value; (**b**) proportion in $\bar{F}$.

Figure 13a presents the absolute values of ${\bar{F}}_{s}$, ${\bar{F}}_{f}$, and ${\bar{F}}_{c}$, while their respective proportions in $\bar{F}$ are illustrated in Figure 13b. Throughout this section, the shell-wall thickness and material of the FFCSCSs remain constant, ensuring that ${\bar{F}}_{s}$ remains consistent for each FFCSCS. In Figure 13a, as ${\bar{\rho }}_{f}$ increases, both ${\bar{F}}_{f}$ and ${\bar{F}}_{c}$ initially increase, followed by a subsequent decrease for each structure. In Figure 13b, it can be observed that when ${\bar{\rho }}_{f}$ is equal to 0.6, ${\bar{F}}_{s}$ accounts for the highest proportion (82%), followed by ${\bar{F}}_{c}$ (16%), and ${\bar{F}}_{f}$ represents the lowest proportion (2%). As ${\bar{\rho }}_{f}$ increases, the proportion of ${\bar{F}}_{s}$ decreases, while the proportions of ${\bar{F}}_{f}$ and ${\bar{F}}_{c}$ increase. This observation indicates that the contribution of the foam itself and the coupling effects in energy absorption grow with increasing foam density. However, when ${\bar{\rho }}_{f}$ reaches 0.19, the structure undergoes global deformation, resulting in a reduction in the coupling effect and subsequently a decrease in the proportion of ${\bar{F}}_{c}$ in $\bar{F}$.

### 5.2. Influence of Shell-Wall Thickness on the Coupling Effect

This section examines the influence of the wall thickness (*t*) on the coupling strengthening effect in FFCSCSs. Three distinct wall thicknesses are considered: 0.6 mm, 0.8 mm, and 1.0 mm, respectively. For the structures discussed in this section, 6063 Al is employed for both the corrugated core and face sheets while maintaining the relative foam density within the range of 0.06 to 0.19.

**Figure 14:**
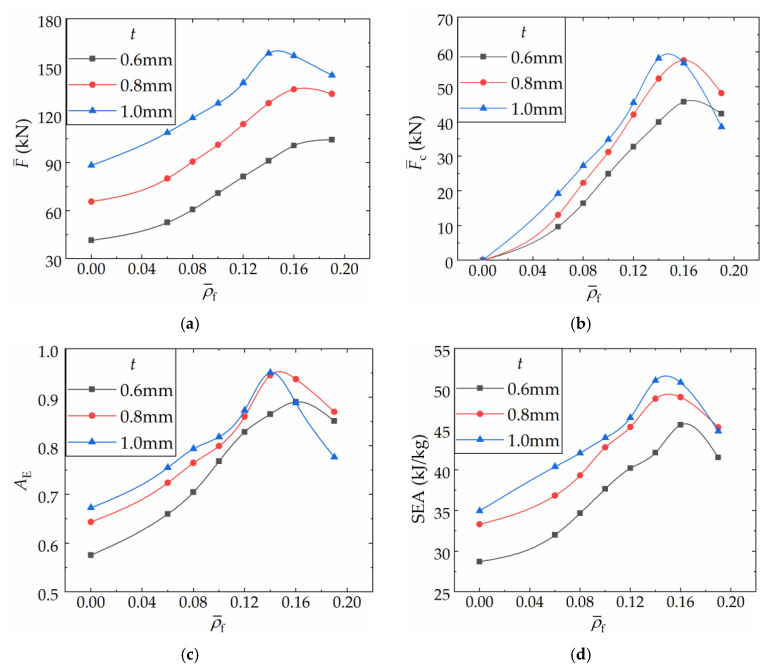
Influence of wall thickness and relative density of foam on the crushing properties of FFCSCSs: (**a**) mean crushing force, $\bar{F}$ (**b**) coupling mean crushing force, ${\bar{F}}_{c}$; (**c**) crushing force efficiency, *A*_E_; (**d**) specific energy absorption, SEA.

Figure 14 illustrates the influence of shell-wall thickness on the crushing performance and coupling strengthening effect of FFCSCSs, considering a range of ${\bar{\rho }}_{f}$ values from 0 to 0.19. It is important to note that ${\bar{\rho }}_{f}=0$ represents the CSCS structure. The results clearly demonstrate that the FFCSCSs exhibit higher values of $\bar{F}$, ${\bar{F}}_{c}$, *A*_E_, and SEA compared to the CSCSs. For a given value of *t*, as ${\bar{\rho }}_{f}$ increases before global deformation occurs, there is a corresponding increase in $\bar{F}$, ${\bar{F}}_{c}$, *A*_E_, and SEA. However, when the value of ${\bar{\rho }}_{f}$ exceeds a certain threshold, global deformation occurs, leading to a decrease in $\bar{F}$, ${\bar{F}}_{c}$, *A*_E_, and SEA. Furthermore, for a given value of ${\bar{\rho }}_{f}$, prior to the occurrence of global deformation, higher values of *t* are associated with increased values of $\bar{F}$, ${\bar{F}}_{c}$, *A*_E_, and SEA.

Moreover, as illustrated in Figure 14, the critical threshold of ${\bar{\rho }}_{f}$ at which FFCSCSs undergo global deformation varies depending on the values of *t*. A higher *t* value is associated with a lower critical threshold of ${\bar{\rho }}_{f}$. This finding indicates that structures with thicker walls are more prone to global deformation. Furthermore, once the structure undergoes global deformation, a higher value of *t* results in a more substantial decline in $\bar{F}$, ${\bar{F}}_{c}$, *A*_E_, and SEA. To illustrate this, let us consider ${\bar{F}}_{c}$ as an example. When *t* values are set at 0.6 mm, 0.8 mm, and 1.0 mm, the corresponding reductions in ${\bar{F}}_{c}$ during global deformation are 8%, 15%, and 28%, respectively. Overall, FFCSCSs exhibit superior energy absorption capabilities compared to CSCSs. Notably, FFCSCSs with greater wall thicknesses demonstrate a pronounced coupling strengthening effect, resulting in higher energy absorption capacities.

**Figure 15:**
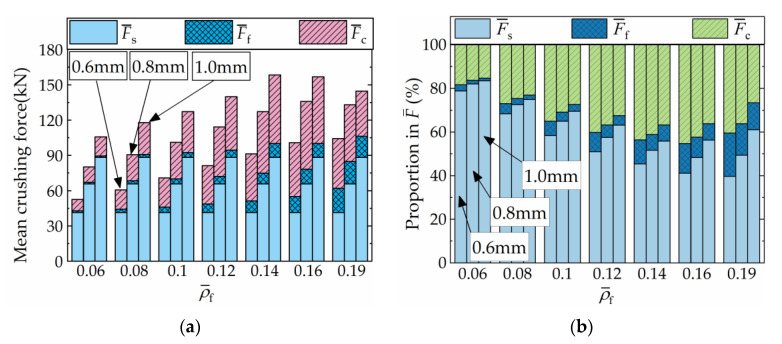
Composition of the mean crushing force for FFSCSCs with different wall thicknesses and relative densities of foam: (**a**) absolute value; (**b**) proportion in $\bar{F}$.

Figure 15 illustrates the absolute values of ${\bar{F}}_{s}$, ${\bar{F}}_{f}$, and ${\bar{F}}_{c}$, as well as their respective proportions in $\bar{F}$, for various combinations of *t* and ${\bar{\rho }}_{f}$. In Figure 15a, when a specific value of ${\bar{\rho }}_{f}$ is considered, the bar charts represent ${\bar{F}}_{s}$, ${\bar{F}}_{f}$ and, ${\bar{F}}_{c}$ for FFCSCSs with different *t* values (0.6 mm, 0.8 mm, and 1.0 mm), arranged from left to right. It is evident that when a specific value of ${\bar{\rho }}_{f}$ is provided, ${\bar{F}}_{s}$ exhibits an increasing trend as *t* increases. In the case of ${\bar{F}}_{f}$, as *t* increases, the foam-filled area within the corrugated channel decreases, resulting in a reduction in ${\bar{F}}_{f}$. However, within the discussed range, the differences in *t* values are relatively small, resulting in less noticeable variations in ${\bar{F}}_{f}$ for different *t* values. For ${\bar{F}}_{c}$, before the global deformation occurs, a larger *t* corresponds to a larger ${\bar{F}}_{c}$ for the same ${\bar{\rho }}_{f}$. Additionally, when *t* is held constant, an increase in ${\bar{\rho }}_{f}$ results in no change in ${\bar{F}}_{s}$, while ${\bar{F}}_{f}$ and ${\bar{F}}_{c}$ increase. In Figure 15b, for a specific value of ${\bar{\rho }}_{f}$, the bar charts, from left to right, represent the proportions of ${\bar{F}}_{s}$, ${\bar{F}}_{f}$, and ${\bar{F}}_{c}$ in $\bar{F}$ for *t* values of 0.6 mm, 0.8 mm, and 1.0 mm, respectively. It is evident that when a specific value of ${\bar{\rho }}_{f}$ is given, an increase in *t* results in a higher proportion of ${\bar{F}}_{s}$, while the proportions of ${\bar{F}}_{f}$ and ${\bar{F}}_{c}$ decrease. Conversely, when *t* is held constant, before global deformation occurs, the proportion of ${\bar{F}}_{s}$ decreases, and the proportion of ${\bar{F}}_{f}$ and ${\bar{F}}_{c}$ increases as ${\bar{\rho }}_{f}$ increases.

It is observed that, prior to the occurrence of global deformation, higher relative foam density and greater shell-wall thickness contribute to a strengthened coupling effect among the structural components. Conversely, greater relative foam density and smaller shell-wall thickness result in an increased proportion of the coupling strengthening effect in structural energy dissipation.

### 5.3. Influence of Shell Material on the Coupling Effect

This section investigates the influence of shell materials in FFCSCSs on the coupling strengthening effect. Three different materials are considered: 6063 Al, 6061 Al, and 304L stainless steel. Among these materials, there is a gradual increase in both yield stress and flow stress, progressing from 6063 Al to 6061 Al and finally to 304L stainless steel. In the considered structure, the corrugated core and face sheet wall thickness *t* is fixed at 0.8 mm, while the relative density of the foam ${\bar{\rho }}_{f}$ ranges from 0.06 to 0.19.

**Figure 16:**
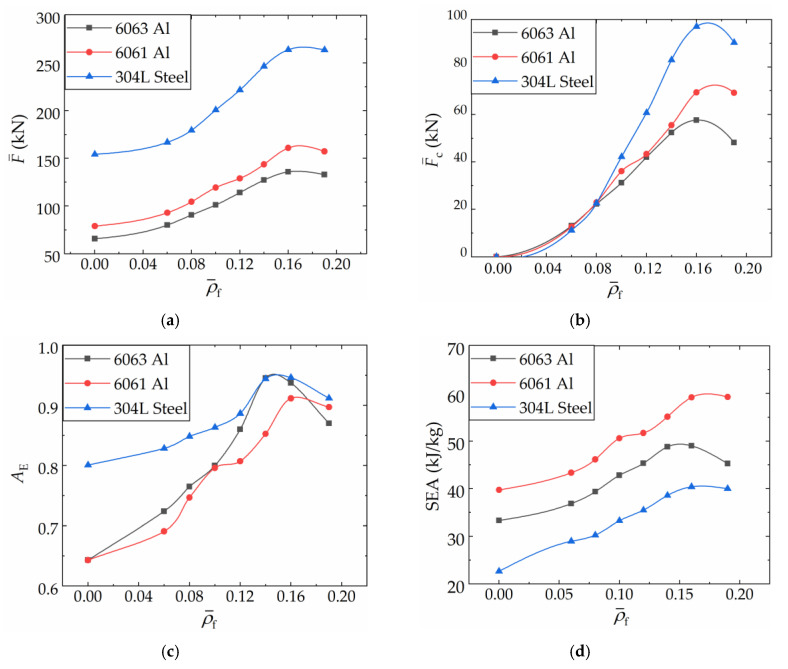
Influence of wall material and relative density of foam on the crushing properties of FFCSCs: (**a**) mean crushing force, $\bar{F}$; (**b**) coupling mean crushing force, $\bar{F}$; (**c**) crushing force efficiency, *A*_E_; (**d**) specific energy absorption, SEA.

Figure 16 illustrates the impact of shell material on the crushing performance and coupling strengthening effect of FFCSCSs. It is observed that all FFCSCSs exhibit higher values for $\bar{F}$, ${\bar{F}}_{c}$, *A*_E_, and SEA compared to the CSCSs. For each material, as the relative density ${\bar{\rho }}_{f}$ increases up to 0.16, $\bar{F}$, ${\bar{F}}_{c}$, *A*_E_, and SEA increase accordingly. However, when ${\bar{\rho }}_{f}$ exceeds 0.16, the structure experiences global deformation, resulting in a decrease in energy absorption performance and a subsequent decline in $\bar{F}$, ${\bar{F}}_{c}$, *A*_E_, and SEA.

Furthermore, for a given ${\bar{\rho }}_{f}$, the performance of the structure is significantly influenced by the strength of the shell material, with higher strength materials exhibiting greater values for $\bar{F}$, ${\bar{F}}_{c}$, and *A*_E_. However, in Figure 16d, it is observed that for the same ${\bar{\rho }}_{f}$, the SEA of the 6061 Al structure is the highest, followed by the 6063 Al, while the 304L stainless steel exhibited the lowest SEA. The reason for this phenomenon can be analyzed as follows. Referring to Figure 4, although the flow stress of 304L stainless steel is 1.9 times that of the 6063 Al, its density is 2.9 times that of the 6063 Al as well, resulting in a lower SEA. On the other hand, both 6063 Al and 6061 Al have the same density, but the 6061 Al has higher yield stress and flow stress compared to the 6063 Al, leading to a higher SEA for the 6061 Al structures. Overall, the FFCSCSs consistently demonstrate superior energy absorption performance compared to the CSCSs for all materials considered. The coupling strengthening effect and mean crushing force of the FFCSCSs are strengthened with higher flow stress in the shell material. The specific energy absorption of the structure is influenced by both the flow stress and the density of the base material.

**Figure 17:**
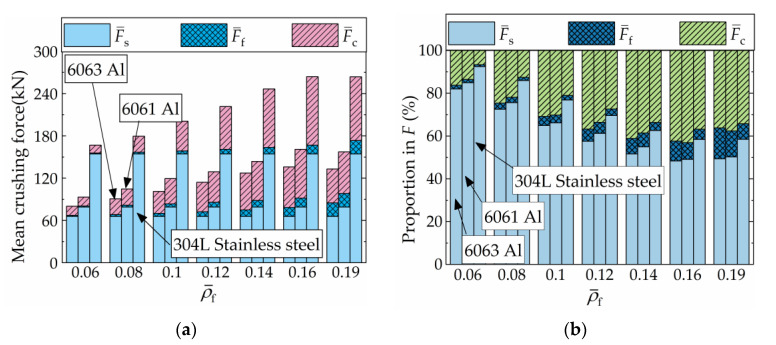
Composition of the mean crushing force for FFSCSCs with different wall materials and relative densities of foam: (**a**) absolute value; (**b**) proportion in $\bar{F}$.

Figure 17 provides an analysis of the absolute values of ${\bar{F}}_{s}$, ${\bar{F}}_{f}$, and ${\bar{F}}_{c}$ with different shell materials and ${\bar{\rho }}_{f}$, along with their respective proportions in $\bar{F}$. In Figure 17a, for a specific value of ${\bar{\rho }}_{f}$, the bar charts depict ${\bar{F}}_{s}$, ${\bar{F}}_{f}$, and ${\bar{F}}_{c}$ for the FFCSCSs with different shell materials (6063 Al, 6061 Al, and 304L stainless steel), arranged in ascending order of material flow stress from left to right. It is observed that when a specific ${\bar{\rho }}_{f}$ value is assigned, both ${\bar{F}}_{s}$ and ${\bar{F}}_{c}$ increase with an increase in material flow stress, while ${\bar{F}}_{f}$ remains constant. Similarly, for a given material, as ${\bar{\rho }}_{f}$ increases before global deformation occurs, ${\bar{F}}_{s}$ remains constant, while both ${\bar{F}}_{f}$ and ${\bar{F}}_{c}$ increase. In Figure 17b, when ${\bar{\rho }}_{f}$ is assigned, the bar charts from left to right represent the proportion of ${\bar{F}}_{s}$, ${\bar{F}}_{f}$, and ${\bar{F}}_{c}$ in $\bar{F}$ for 6063 Al, 6061 Al, and 304L stainless steel, respectively. With a constant ${\bar{\rho }}_{f}$ value, an increase in material flow stress results in a higher proportion of ${\bar{F}}_{s}$, accompanied by a lower proportion of ${\bar{F}}_{c}$ and ${\bar{F}}_{f}$. Likewise, when a specific material is given, before the structure undergoes global deformation, the proportion of ${\bar{F}}_{s}$ decreases with an increasing value of ${\bar{\rho }}_{f}$, while the proportions of ${\bar{F}}_{f}$ and ${\bar{F}}_{c}$ increase.

Consequently, an increase in foam relative density and shell material strength results in a stronger coupling strengthening effect among the components of FFCSCSs. Conversely, higher foam relative density and weaker shell material lead to a greater proportion of the coupling strengthening effect in energy absorption.

## 6. Theoretical Analysis

Based on the findings above, it is evident that the coupling strengthening effect increases with higher foam density, greater wall thickness of the shells, and higher flow stress of the shell material. In this section, a theoretical model is derived for predicting the mean crushing force of the FFCSCSs. The development of this model builds upon our previous work [54] for predicting the mean crushing force of CSCSs and incorporates insights regarding the coupling effect of foam-filled square tube structures [69].

### 6.1. Theoretical Model

According to Equation (2), the mean crushing force ($\bar{F}$) of the FFCSCS is determined as the sum of the mean crushing forces of the shell (${\bar{F}}_{s}$) and foam core (${\bar{F}}_{f}$) and the coupling contribution (${\bar{F}}_{c}$). The calculation of ${\bar{F}}_{s}$ is based on our former theoretical model, the detailed solution process of which can be referred to in Ref. [54]. During the solution process, the energy absorption of each folded cell within a folding cycle of 2*H* is cumulatively calculated. Based on the principle of energy balance (which ensures that the work performed by external forces is equal to the internal energy dissipation), the expression for ${\bar{F}}_{s}$ is derived as follows:

(12) $${\bar{F}}_{s}=W_{\mathrm{total}}/2H\xi$$

where *W*_total_ is determined as the function in terms of *H* and *b*. Here, *b* refers to the radius of the toroidal surface in the super folding elements (not shown for brevity), while *H* represents the half-length of the fold. ξ denotes the effective crush distance coefficient.

The actual crushing mode of FFCSCSs should minimize the mean crushing force [5]. Therefore, it is crucial to ensure that

(13) $$\begin{array}{cc} \frac{\partial {\bar{F}}_{s}}{\partial H}=0 & \frac{\partial {\bar{F}}_{s}}{\partial b}=0 \end{array}$$

After solving the aforementioned equation, the resulting values of *H* and *b* are then used in Equation (8) to calculate ${\bar{F}}_{s}$.

The foam mean crushing force ${\bar{F}}_{f}$ can be calculated from [69]:

(14) $${\bar{F}}_{f}=\sigma_{f}S_{\mathrm{foam}}$$

where *σ*_f_ is defined as the plateau stress of foam when compressed to 50%, and *S*_foam_ represents the cross-sectional area of the foam perpendicular to the compression direction.

To compute *σ*_f_ for foams with a range of ${\bar{\rho }}_{f}$ values spanning from 0.05 to 0.2, Equation (5) can be applied as follows:

(15) $$\sigma_{f}=2\int_{0}^{0.5}\left\{\sigma_{p}+42{\bar{\rho }}_{f}{}^{1.42}\frac{e}{e_{D}}+251{\bar{\rho }}_{f}\ln\left[\frac{1}{1-{\left(e/e_{D}\right)}^{\beta }}\right]\right\}de$$

According to reference [69], the general expression of the coupling mean crushing force ${\bar{F}}_{c}$ is provided as follows:

(16) $${\bar{F}}_{c}=NC_{\mathrm{avg}}\sigma_{f}^{\alpha }\sigma_{o}^{\left(1-\alpha \right)}w_{}^{\beta }t^{\left(2-\beta \right)}$$

where *N* represents the number of corrugated cells. *C*_avg_, *α*, and *β* are dimensionless parameters that describe the coupling strengthening effect. The equation involves the plateau stress of foam *σ*_f_, the flow stress of the shell material *σ*_o_, the width of the corrugated core *w*, and the thickness of shell walls *t*.

Thus, the mean crushing force $\bar{F}$ can be expressed as follows:

(17) $$\bar{F}={\bar{F}}_{s}+\sigma_{f}S_{\mathrm{foam}}+NC_{\mathrm{avg}}\sigma_{f}^{\alpha }\sigma_{o}^{\left(1-\alpha \right)}w_{}^{\beta }t^{\left(2-\beta \right)}$$

The first two terms in Equation (13) can be computed directly from the geometric and material parameters of FFCSCSs. However, the third term, representing the coupling mean crushing force, depends on three dimensionless parameters: *C*_avg_, *α*, and *β*. In reference [69], these dimensionless parameters were obtained through fitting the experimental data. In this study, a similar fitting approach is employed to ascertain the values of *C*_avg_, *α*, and *β*. This is achieved using MATLAB’s built-in multiple nonlinear regression function, “nlinfit”, which is based on numerical simulations. The goodness of fit is assessed using the coefficient of determination *R*^2^ between the theoretical predictions and simulated results. A higher *R*^2^ value, closer to 1, indicates a more reliable and higher-quality fit. The expression for *R*^2^ is provided as follows:

(18) $$R^{2}=1-\frac{\sum_{i=1}^{M}{\left(y_{i}-{\hat{y}}_{i}\right)}^{2}}{\sum_{i=1}^{M}{\left(y_{i}-{\bar{y}}_{i}\right)}^{2}}$$

where $y_{i}$ represents the simulated results, ${\bar{y}}_{i}$ denotes the mean value of simulations, ${\hat{y}}_{i}$ represents the theoretical predictions, and *M* corresponds to the number of fitted samples.

### 6.2. Comparison with Simulated Results

The finite element analysis presented in Section 4 shows that the structure undergoes global deformation when ${\bar{\rho }}_{f}$ exceeds a certain threshold value. Consequently, the theoretical model mentioned above is found to be inapplicable in such cases. Therefore, in developing the theoretical model, only FFCSCSs with ${\bar{\rho }}_{f}$ values ranging from 0.06 to 0.14 were considered. Based on the simulated results, through multivariate nonlinear regression analysis, the values of *C*_avg_, *α*, and *β* were determined as 6.6051, 0.6796, and 1.3236, respectively. The predicted values were calculated by substituting these values into Equations (13) and (14).

**Figure 18:**
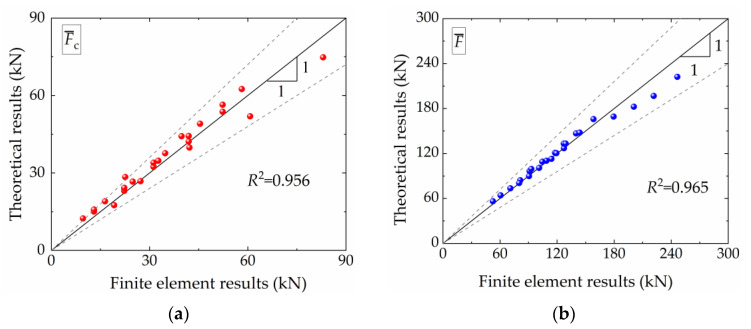
Comparison of theoretical predicated and finite element results: (**a**) coupling mean crushing force, ${\bar{F}}_{c}$; (**b**) mean crushing force, $\bar{F}$.

Figure 18 displays both the simulated and theoretical results for the mean crushing force $\bar{F}$ and the coupling mean crushing force ${\bar{F}}_{c}$. The *x* axis represents the simulated results, and the *y* axis represents the theoretical predictions. The solid black line (45° diagonal line) represents perfect agreement between the theoretical and simulated results, while the gray dashed line represents an error margin of ±20% between the simulated and theoretical results. In Figure 18a, the theoretical results and corresponding simulated results for ${\bar{F}}_{c}$ are shown. All data points are distributed on both sides of the 45° diagonal line, indicating strong agreement between the theoretical and simulated results. The coefficient of determination *R*^2^, obtained using Equation (15), is 0.956, affirming the reliability of the obtained values of *C*_avg_, *α*, and *β*. Figure 18b displays the theoretical and corresponding simulated results for $\bar{F}$. The agreement between the simulated and theoretical results of $\bar{F}$ is higher compared to ${\bar{F}}_{c}$, with data points more closely aligned to the 45° diagonal line. Additionally, the *R*^2^ value for the predicted value of $\bar{F}$ is 0.965. Consequently, the proposed theoretical model for predicting $\bar{F}$ and ${\bar{F}}_{c}$ within the discussed range of ${\bar{\rho }}_{f}$ values is deemed reliable.

### 6.3. Parametric Studies

In Section 4, the influence of foam relative density ${\bar{\rho }}_{f}$ on the coupling strengthening effect was discussed using the finite element method. However, due to computational limitations and the complexity of numerical models, the compared structures did not adhere to the principle of equal mass, and the interval of ${\bar{\rho }}_{f}$ is relatively large (0.02). In this section, the theoretical model is employed to investigate the influence of ${\bar{\rho }}_{f}$ on $\bar{F}$, ${\bar{F}}_{s}$, ${\bar{F}}_{f}$, and ${\bar{F}}_{c}$ while adhering to the principle of equal mass. To achieve this, the mass of FFCSCSs is equated with that of the CSCS with a wall thickness of *t* = 1 mm. As ${\bar{\rho }}_{f}$ increases, the mass of FFCSCSs remains constant by reducing the value of *t.* In this section, ${\bar{\rho }}_{f}$ varies within the range of 0.6 to 0.14, with a finer interval of 0.002, allowing for a more precise analysis of the influence of ${\bar{\rho }}_{f}$ on the mentioned parameters.

**Figure 19:**
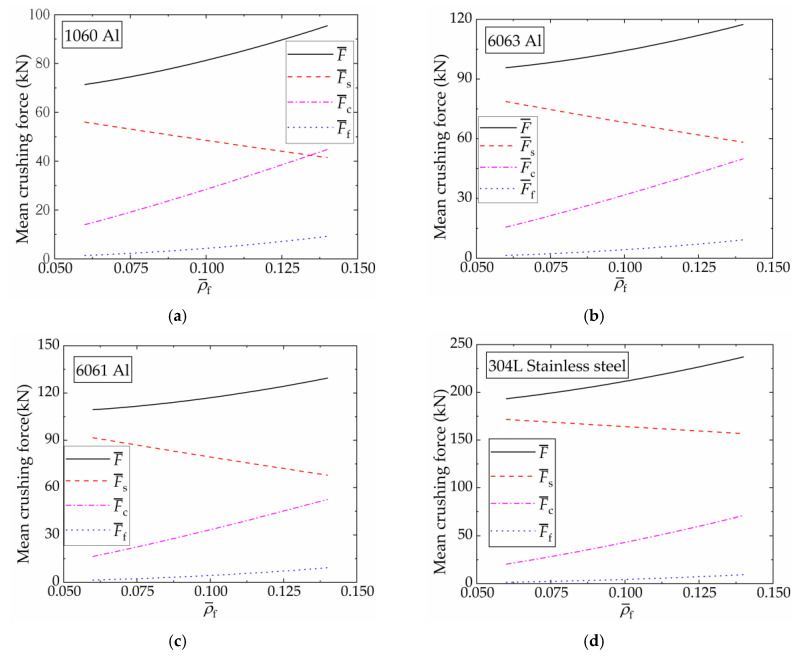
Theoretical predictions of mean crushing force of FFCSCSs with equal mass: (**a**) 1060 Al face sheets with different ${\bar{\rho }}_{f}$; (**b**) 6063 Al face sheets with different ${\bar{\rho }}_{f}$; (**c**) 6061 Al face sheets with different ${\bar{\rho }}_{f}$; (**d**) 304L stainless steel face sheets with different ${\bar{\rho }}_{f}$.

Figure 19 presents the variation characteristics of $\bar{F}$, ${\bar{F}}_{s}$, ${\bar{F}}_{f}$, and ${\bar{F}}_{c}$ in different FFCSCSs with equal mass, as a function of ${\bar{\rho }}_{f}$. The base materials considered here are 1060 Al, 6063 Al, 6061 Al, and 304L stainless steel. The flow stress for 1060 Al is set to 140 MPa, while the values for the other three materials can be found in Section 3.3. Figure 19 shows that for a given shell material, increasing ${\bar{\rho }}_{f}$ necessitates a reduction in *t* to maintain the same structural mass. Throughout this process, $\bar{F}$, ${\bar{F}}_{f}$, and ${\bar{F}}_{c}$ show an increase, while ${\bar{F}}_{s}$ experiences a decrease. Among the FFCSCSs made of 6063 Al, 6061 Al, and 304L stainless steel, the mean crushing force can be arranged in descending order as ${\bar{F}}_{s}$, ${\bar{F}}_{c}$, and ${\bar{F}}_{f}$. Additionally, the difference between ${\bar{F}}_{s}$ and ${\bar{F}}_{s}$ decreases with an increasing ${\bar{\rho }}_{f}$. In the case of the FFCSCSs made of 1060 Al, when ${\bar{\rho }}_{f}$ is less than 0.13, ${\bar{F}}_{s}$ is greater than both ${\bar{F}}_{c}$ and ${\bar{F}}_{f}$. However, when ${\bar{\rho }}_{f}$ exceeds 0.13, ${\bar{F}}_{s}$ becomes smaller than ${\bar{F}}_{c}$. In general, based on equal mass, $\bar{F}$ increases with ${\bar{\rho }}_{f}$, corresponding to the increased energy absorption. Simultaneously, ${\bar{F}}_{s}$ decreases, while ${\bar{F}}_{f}$ and ${\bar{F}}_{c}$ increase.

**Figure 20:**
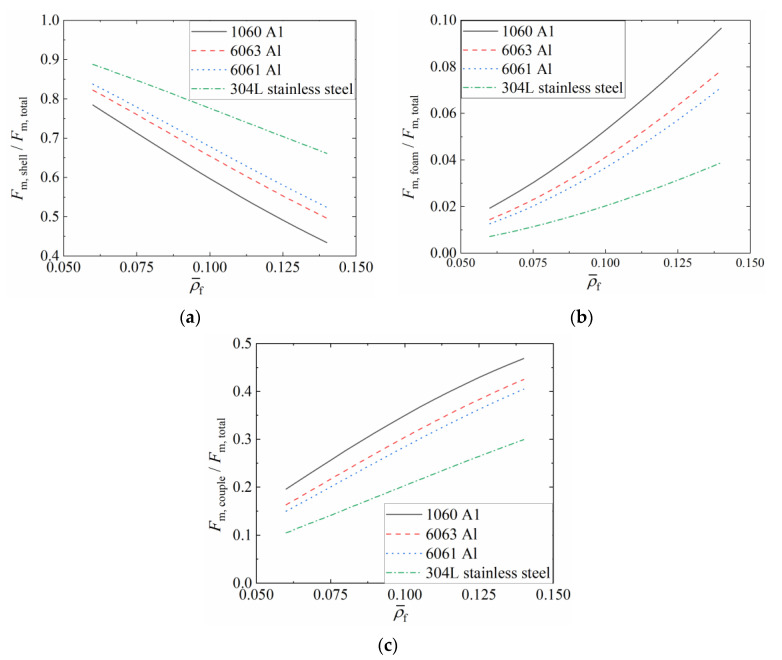
Proportion of mean crushing force of FFSCSCs: (**a**) ${\bar{F}}_{s}$; (**b**) ${\bar{F}}_{f}$; (**c**) ${\bar{F}}_{c}$.

Figure 20 presents the variation characteristics of the proportion of ${\bar{F}}_{s}$, ${\bar{F}}_{f}$, and ${\bar{F}}_{c}$ in $\bar{F}$ under equal mass conditions as a function of ${\bar{\rho }}_{f}$. Figure 20a shows the proportional contribution of ${\bar{F}}_{s}$ in $\bar{F}$. For a given material, the proportion of ${\bar{F}}_{s}$ decreases as ${\bar{\rho }}_{f}$ increases. Conversely, for a given ${\bar{\rho }}_{f}$, higher material flow stress results in a more significant proportion of ${\bar{F}}_{s}$ in $\bar{F}$. Figure 20b displays the proportional contribution of ${\bar{F}}_{f}$ in $\bar{F}$. It can be observed that for a given material, the proportion of ${\bar{F}}_{f}$ increases with increasing ${\bar{\rho }}_{f}$, while for a given ${\bar{\rho }}_{f}$, higher material flow stress leads to a lower proportion of ${\bar{F}}_{f}$. Figure 20c demonstrates the proportional contribution of ${\bar{F}}_{c}$ in $\bar{F}$. It can be seen that for a given material, the proportion of ${\bar{F}}_{c}$ increases as ${\bar{\rho }}_{f}$ increases, whereas for a given ${\bar{\rho }}_{f}$, higher material flow stress results in a lower proportion of ${\bar{F}}_{c}$ in $\bar{F}$.

In conclusion, based on equal mass conditions, a higher ${\bar{\rho }}_{f}$ contributes to greater energy absorption in FFCSCSs. This contribution can be attributed to both the foam itself and the coupling strengthening effect. When ${\bar{\rho }}_{f}$ is held constant, a higher shell flow stress leads to a larger energy absorption, with a significant contribution from the shell itself but a smaller contribution from the coupling effect and foam.

## 7. Conclusions

The concept of material hybrid design was introduced to incorporate aluminum foam into the corrugated channels of the corrugated core sandwich cylindrical shell, thereby creating a novel foam-filled corrugated sandwich cylindrical shell (FFCSCSs). The energy absorption characteristics of FFCSCSs were systematically investigated through a combination of simulations and theoretical analysis. The main conclusions are summarized as follows:

1. The FFCSCS demonstrates significantly enhanced energy absorption performance under axial compression, primarily due to the foam filling, resulting in maximum specific energy absorption of 60 kJ/kg. Furthermore, the coupling strengthening effect is notably pronounced, as evidenced by the maximum value of ${\bar{F}}_{c}/\bar{F}$, which reaches up to 40%.
2. The coupling strengthening effect is primarily observed in two aspects. Firstly, the intrusion of folds into the foam leads to a more comprehensive compression of the foam insertions. Secondly, influenced by foam insertions, the folds bend along the compression direction and compress against each other, thereby expanding the plastic deformation zone.
3. In FFCSCSs, as the foam relative density, shell-wall thickness, and material flow stress increase, the coupling strengthening effect among the components strengthens, resulting in improved energy absorption performance, enhanced crushing efficiency, and increased mean crushing force.
4. The theoretical predictions strongly agree with the results of the finite element simulations. A parametric analysis based on the theoretical model shows that an increase in foam density leads to an increase in $\bar{F}$. Simultaneously, the proportion of ${\bar{F}}_{s}$ decreases, while the proportions of ${\bar{F}}_{f}$ and ${\bar{F}}_{c}$ increase.

## Figures and Tables

**Table 1:** Comparison of energy absorption of FFCSCS 6063-08-01 and its individual components.

Structures	$\bar{F}$		${\bar{F}}_{c}$		SEA (kJ/kg)	*A*_E_
	Value (kN)	Percentage	Value (kN)	Percentage		
CSCS	65.7	65%	/	/	33.38	0.64
Foam	4.29	4%	/	/	24.79	/
FFCSCS	101.19	/	31.2	31%	42.79	0.8

**Table 2:** Energy absorption of each component in FFCSCS, CSCS and the foam.

Component	FFCSCS		CSCS		Foam (kJ)	*E* Enhancement
	Value (kJ)	Percentage	Value (kJ)	Percentage		
Outer face	1.2	25%	1.056	30%	/	14%
Inner face	0.96	20%	0.76	22%	/	26%
Corrugation	1.76	36%	1.68	48%	/	5%
Foam	0.92	19%	/	/	0.24	283%

**Table 3:** Comparison of collapse configuration of each component in the FFCSCS and CSCS.

	CSCS	FFCSCS
IF	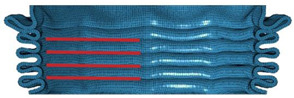	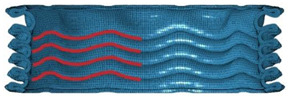
OF	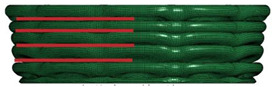	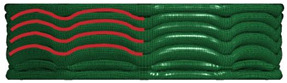
Core	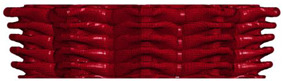	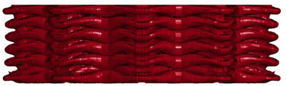

**Table 4:** Crushing properties of FFSCSCs with ${\bar{\rho }}_{f}=0\sim 0$.019.

${\bar{\rho }}_{f}$	$\bar{F}$ (kN)	${\bar{F}}_{c}$ (kN)	SEA (kJ/kg)	*A*_E_
0	65.70	/	33.30	0.64
0.06	80.11	13.03	36.85	0.72
0.08	90.59	22.28	39.34	0.81
0.10	101.19	31.20	42.79	0.80
0.12	114.14	41.97	45.32	0.86
0.14	127.25	52.34	48.80	0.99
0.16	135.91	57.62	49.00	0.94
0.19	132.99	48.15	45.26	0.87

## Data Availability

The data presented in this study are available on request from the references and the corresponding author.

## Nomenclature

*H*_s_	initial height of cylindrical shell
*R*_o_	outer radius of the sandwich cylindrical shell
*R*_i_	inter radius of the sandwich cylindrical shell
*t*	thickness of the face sheets and corrugated core
*w*	width of the corrugated cell
*N*	number of the corrugated core
$\rho_{f}$	density of the filling foam
${\bar{\rho }}_{f}$	relative density of the filling foam
$\sigma_{o}$	flow stress of the metal material
*e*	engineering strain of the foam
*e*_D_	engineering compaction strain of the foam
$\sigma_{p}$	yield strength of the foam
*E*_p_	modulus of elasticity of the foam
*d*	compressing displacement
*F*	crushing force
*F*_max_	maximal value of F during compression
$\bar{F}$	mean crushing force of the entire structure
${\bar{F}}_{s}$	mean crushing force of the corrugated sandwich cylindrical shell
${\bar{F}}_{f}$	mean crushing force of the filling foam
${\bar{F}}_{c}$	mean crushing force sourced from the coupling effect
*E*	energy absorption by the structure
*A*_E_	crushing force efficiency
*T*_E_	energy absorption efficiency
SEA	specific energy absorption
*H*	half-length of the fold
*b*	radius of toroidal surface in the super folding elements
ξ	effective crush distance coefficient
*C*_avg_, *α* and *β*	dimensionless parameters which described the coupling enhancement effect
